# Enzymatic Approach in Calcium Phosphate Biomineralization: A Contribution to Reconcile the Physicochemical with the Physiological View

**DOI:** 10.3390/ijms222312957

**Published:** 2021-11-30

**Authors:** Clément Guibert, Jessem Landoulsi

**Affiliations:** 1 Sorbonne Université, CNRS, Laboratoire de Réactivité de Surface, LRS, F-75005 Paris, France; 2 Université de Technologie de Compiègne, CNRS, Laboratoire de Biomécanique & Bioingénierie, F-60205 Compiègne, France

**Keywords:** biomineralization, calcium phosphate, collagen, matrix vesicles, enzymes

## Abstract

Biomineralization is the process by which organisms produce hard inorganic matter from soft tissues with outstanding control of mineral deposition in time and space. For this purpose, organisms deploy a sophisticated “toolkit” that has resulted in significant evolutionary innovations, for which calcium phosphate (CaP) is the biomineral selected for the skeleton of vertebrates. While CaP mineral formation in aqueous media can be investigated by studying thermodynamics and kinetics of phase transitions in supersaturated solutions, biogenic mineralization requires coping with the inherent complexity of biological systems. This mainly includes compartmentalization and homeostatic processes used by organisms to regulate key physiological factors, including temperature, pH and ion concentration. A detailed analysis of the literature shows the emergence of two main views describing the mechanism of CaP biomineralization. The first one, more dedicated to the study of in vivo systems and supported by researchers in physiology, often involves matrix vesicles (MVs). The second one, more investigated by the physicochemistry community, involves collagen intrafibrillar mineralization particularly through in vitro acellular models. Herein, we show that there is an obvious need in the biological systems to control both where and when the mineral forms through an in-depth survey of the mechanism of CaP mineralization. This necessity could gather both communities of physiologists and physicochemists under a common interest for an enzymatic approach to better describe CaP biomineralization. Both homogeneous and heterogeneous enzymatic catalyses are conceivable for these systems, and a few preliminary promising results on CaP mineralization for both types of enzymatic catalysis are reported in this work. Through them, we aim to describe the relevance of our point of view and the likely findings that could be obtained when adding an enzymatic approach to the already rich and creative research field dealing with CaP mineralization. This complementary approach could lead to a better understanding of the biomineralization mechanism and inspire the biomimetic design of new materials.

## 1. Introduction

**Figure 1:**
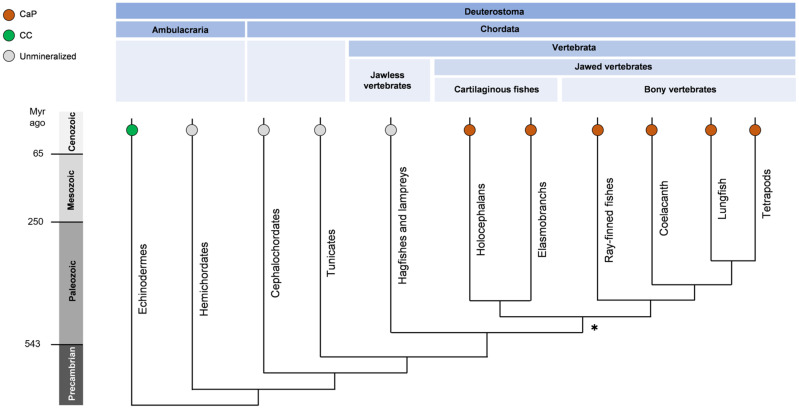
A simplified phylogenetic tree of deuterostomia showing the composition of animal skeletons (CaP: calcium phosphate; CC: calcium carbonate, or unmineralized). Bony vertebrates and cartilaginous fishes are the dominating living vertebrates (gnathostomes, 99.9%). Cartilaginous fishes produce dermal skeleton, including teeth dermal denticle and fin spine, but not endochondral bone as bony vertebrates. Lampreys and hagfishes (cyclostomes) lack, however, mineralized tissues. The emergence of hinged jaws and the mineralized skeleton, indicated by (*), is a major event in the evolution of vertebrates. Adapted from [6]. The composition of the mineral phase was found in [5].

Mineralized cartilage, bones and teeth (dentin and enamel) are tissues unique to vertebrates and are among the main evolutionary innovations that have driven considerable diversification of these animals [1]. The emergence of hinged jaws marks a major event in the evolution of vertebrates (Figure 1), resulting in the dominance of Gnathostomes (99.9% of living vertebrates) [2]. The latter are divided into two groups, which diverged about 422–463 million years (Myr) ago [3]: (i) cartilaginous fishes (Chondrichthyes) and (ii) bony vertebrates (Euteleostomi). The animals of the first group possess principally cartilaginous endoskeletons but also produce dermal bone, such as teeth, dermal denticle and fin spine, as well as mineralized cartilage. By contrast, bony vertebrates have ossified skeletons and have the ability to replace the cartilage with endochondral bone. How these evolutionary innovations emerged and how they resulted in the different mineralized tissues are key questions that are not completely clarified. One important hypothesis suggests that deuterostome, the ancestor of vertebrates and echinoderms, has explored a common way to produce minerals through a specific “biomineralization toolkit” [4]. In [5], Murdock summarized this hypothesis as “the evolution of biomineralization through the co-option of an inherited organic skeleton and genetic toolkit followed by the stepwise acquisition of more complex skeletal tissues under tighter biological control”. The concept of a “biomineralization toolkit” suggests, thus, the deployment of a sophisticated biological system able to control the formation of a mineral phase in terms of composition, structure, morphology and spatial organization within the tissues.

The resulting mineralized tissues are composites made of entangled biological soft matter and inorganic hard mineral, more or less ordered within a variety of hierarchical multi-scale structures. The composition of the mineral phase greatly diverges in the deuterostome super phylum to chordates (more specifically Vertebrata/Gnathostomes) and echinoderms (Figure 1): their skeletons are, respectively, constituted essentially of calcium phosphate (CaP) and calcium carbonate (CC). While the latter had been used to form the exoskeletons of various marine animals for millions of years, the former was selected for the skeleton of vertebrates. The driving factors in this selection have been discussed in the literature [7,8,9] and seem to be related to the lower solubility of CaP in physiological conditions compared to CC phases [10]. Moreover, this provides a source of phosphate that may improve the ability to generate ATP involved in the energy metabolism [8]. Indeed, recent evidence supports the involvement of the skeleton in the endocrine regulation of energy metabolism [11]. The hierarchical structure is a key feature in CaP-mineralized tissues, which is now commonly described according to multiple multiscale levels. Even Vertebrata/Gnathostomes share some common principles in mineral shaping, a great diversification is observed in the structure and function of their skeletons. This remarkable diversity is obvious when describing the skeletons of cartilaginous fishes [12], mammals [13] and Cetacea, such as whales, which exhibit giant, highly porous and lipid-rich bone [14]. The inherent hierarchical structures of these mineralized tissues are at the origin of their outstanding mechanical properties. While the stiffness may increase with the mineral content, as observed in compact bones of several vertebrates [15], mechanical properties are essentially adapted by the modification of the hierarchical structure. This is what is observed, for instance, for energy dissipation due to structural heterogeneities [16] or for compressive properties due to both extra- and intrafibrillar mineralization [17].

The mechanism of biogenic CaP mineralization is complex in vivo. It is mainly due to the inherent characteristics of the biological environment, particularly the structure (compartmentalization) and the dynamics of the regulation processes (homeostasis). CaP mineralization involves a variety of chemical and physiological processes that raise a number of important issues, which have been the subject of numerous and various articles. Two disparate views have emerged in independent ways. The first view describes the involvement of matrix vesicles (MVs) in CaP mineralization and particularly focuses on physiological factors and biochemical processes. The second view describes the mechanism of intrafibrillar mineralization with a particular attention to the role of what is called non-collagenous proteins (NCPs).

Both views have attracted the attention of two noticeably different communities of scientists:

- Researchers in physiology, who focus on shedding light on the mechanisms at stake during the biomineralization in living systems, particularly by exploring the gene knockout approach, and who characterize in vivo systems as precisely as possible.
- Researchers in physicochemistry who are interested in describing the biomineralization process through relevant in vitro models that do not involve cells: they try to take into account the complexity of the systems by selecting and studying a few biological parameters that they expect to play important roles in the process.

This paper aims to point out particular aspects, either experimental or conceptual, that are of primary importance to understanding the chemical and biochemical processes involved in CaP biomineralization. A detailed analysis of the different hypotheses reported in the literature clearly indicates the strong involvement of enzyme-catalyzed reactions in regulating biomineralization processes, obviously in association with other biomacromolecules. Herein, we show the benefits of using an enzymatic approach to investigate the mechanism of CaP mineralization. Considering these aspects is essential to making progress in reconciling disparate physicochemical and physiological views with the aim to decipher the mechanism of CaP biomineralization. This biomimetic system also offers a powerful yet straightforward way for designing advanced materials according to bottom-up approaches while tailoring their properties and functions, with potential use in the repair of mineralized tissues: bone, calcified cartilage, dentin and enamel.

## 2. Mineralization from Supersaturated Solutions

### 2.1. CaP Mineralization Process

The precipitation of CaP from supersaturated solutions in abiotic media has been extensively studied in the literature since the emergence of what is now called classical nucleation theory (CNT). When studying phase transitions in heterogeneous equilibrium in 1876, Gibbs was the first to assess that the stability of a phase is associated with the work required to create a critical nucleus of the new phase [18]. The importance of this work for nucleation was highlighted several years later in the work of Volmer and Webber, who laid the foundations of CNT [19]. More recently, the relevance of CNT in the formation mechanism of CaP minerals has been questioned, and “non-classical” pathways have emerged, which involve, for instance, pre-nucleation clusters of ions [20]. The investigations for CaP precipitation have been conducted under near-physiological conditions (pH, ionic strength (IS) and T) from solutions containing calcium and orthophosphate ions at different supersaturations, mostly exceeding physiological ones (for a review, see [21]). These systems are mainly explored by either monitoring the concentrations of ion precursors in the solution, characterizing the obtained solids (morphology, composition, crystallinity), or a combination of both approaches.

All these studies have led to the elaboration of a body of knowledge regarding the mechanisms of CaP nucleation and growth from supersaturated solutions. A large consensus in the literature is that the in vitro formation of hydroxyapatite (HAP) at 37 °C and physiological pH involves a first step where an amorphous calcium phosphate (ACP) appears at the beginning of the precipitation [22,23]. In 1974, Betts and Posner proposed that this calcium-deficient (compared to HAP) amorphous phase exhibits a short-range order and could, in fact, be composed of small subunits (9.5 Å in diameter) [24], referred to as *Posner’s clusters* (Ca_9_(PO_4_)_6_). Numerous authors link their findings to this concept of small building clusters and tend to find similar dimensions as the one determined originally by X-ray diffraction by Betts and Posner [20,25,26].

ACP is then transformed into HAP by global Ca^2+^ and HO^-^ uptake. The reorganization of the mineral structure has been found to go through the formation of another intermediary phase, octacalcium phosphate (OCP), in neutral conditions [27,28]. This OCP phase can be described as “alternating HAP and hydrated layers” [29,30]. It is not observed by all the authors who studied this process, either because it can be difficult to characterize (unstable and thus short-lasting phase, and difficult to distinguish because of its similarities with HAP) or also because each crystallization mechanism path does not necessarily use this phase as an intermediate, depending on the specific conditions of the experiment. In particular, in more acidic solutions, no ACP nor OCP were observed, but another intermediate phase was observed instead, dicalcium phosphate dihydrate (DCPD) [27]. A recent study [31] mixing experiments (progressive introduction of CaCl_2_ at fixed pH and controlled ionic strength, IS) and thermodynamics and kinetics calculations revisited these results and corroborated them with a mechanism that goes through intermediate phases before forming HAP: DCPD only at low IS, DCPD and then OCP at high IS.

According to the classical nucleation theory (CNT) [19], the formation of the crystalline HAP mineral is expected to occur through a stochastic process, resulting in the formation of nuclei that can grow, if a critical size is reached, and evolve to a mature crystal. As detailed above, experiments showed that the first solid that forms is not necessarily the most thermodynamically stable, but rather, the most kinetically accessible. The identification of ACP and other precursor phases during the HAP crystallization process (e.g., OCP and DCPD) is in accordance with the concept of Ostwald rule of stage [32], where most kinetically favorable (often more soluble) phases form first and then evolve towards the most stable (less soluble) phases.

If a dissolution–recrystallization process was first invoked to describe phase transformations, some experimental observations [33] support another pathway, which goes through an internal rearrangement that does not require dissolution. Andersson et al. [25] proposed a hybrid mechanism involving the transfer of Posner’s clusters as growth subunits. However, their findings should be considered warily since they are supported by TEM observations under dry conditions.

These studies are doubtlessly relevant for providing a comprehensive understanding about CaP biomineralization, but they are too simple to take into account the intricacy of biological systems, which is due, in particular, to their rich and various composition and to their compartmentalized nature. Moreover, the various tests performed from supersaturated solutions showed the high dependency of the CaP mineralization process to the protocol used and experimental parameters, including concentrations of ion precursors and physicochemical conditions [24,31,34,35,36].

In order to model the complexity of biological systems, the in vitro models proposed by the various research teams have studied the effects of additional molecules, particularly to mimic the effect of biomacromolecules. Indeed, it can deeply influence the kinetic behavior of the possible intermediate phases, which is a key parameter in the process that is supposed to be ruled by the Ostwald law. For this purpose, some authors have introduced either small biomolecules, typically amino acids and oligopeptides (see [37] and references therein), or macromolecules, including synthetic polyelectrolytes (for a review, see [38]) and proteins [39,40,41], in the mineralization solution, showing their ability to promote/inhibit CaP nucleation and/or growth. In addition to bio(macro)molecules, the effect of inorganic ions, which can be present in the extracellular fluid, has been investigated. This mainly includes ions, such as magnesium (II) ions [42,43,44,45] or zinc (II) ions [44], that have both been shown to inhibit crystal growth. The inhibitive effect of the adsorption of these ions onto the ACP or HAP surface has been evidenced in [46], and a review of the effect of various ions on the mineralization and the structure of CaP can be found in [47].

### 2.2. Factors Influencing CaP Nucleation and Growth

The nucleation and growth of CaP compounds in vivo are influenced by intrinsic and extrinsic factors that can be summarized as follows:

(i) *Intrinsic factors* related to the concentration of calcium and orthophosphate ions, the main precursors of the CaP compounds. The concentrations of ions are maintained in a narrow range by homeostasis, the process through which organisms regulate important physiological factors. Table 1 provides an approximate content and distribution of calcium and phosphorus in the adult human body. It shows that most of the calcium (>99%) is stored in the skeleton, while only small amounts circulate in the body (2.1–2.6 mM), including calcium bound to plasma proteins (e.g., Fetuin [48]) and free calcium ions (1.1–1.3 mM). These levels are generally maintained within a tight range through multiple regulation processes involving bone, intestines and kidneys [49], and they rarely fluctuate more than 5% over time [50]. The intracellular calcium concentrations (about 100 and 0.1 µM in the endoplasmic reticulum and the cytosol, respectively) are about ten orders of magnitude lower than that of the extracellular fluid concentration [9]. This abrupt gradient is maintained via the presence of calcium channels and pumps in the cell membrane via the mitochondrial uptake and via the calcium sequestration in the endoplasmic reticulum [51]. Regarding phosphorus, it is mainly incorporated in the mineral phase (~85%). Outside the skeleton, over 99% of the body content is intracellular. Accordingly, only a small amount of phosphorus is present in the extracellular space, and about 30% of this phosphorus is in the form of phosphate ions and is present at variable concentrations in the plasma (0.8–1.5 mM).

As shown in Table 2, the ionic product (*IP*) corresponding to HAP can be calculated, taking into account the high ionic strength of the biological medium to compute the activity based on the concentrations of calcium and orthophosphate ions. The comparison of the obtained value with the solubility product (*K_sp_*) of HAP reveals that the solution is in a metastable supersaturated state regarding HAP since no spontaneous precipitation is observed under these conditions. This conclusion is also valid if another CaP phase, different from HAP, is considered, such as OCP and DCPD [21,26], or even ACP for which *K_sp_* values have been reported in the literature [26]. Explanations for this metastability are given below in (iii), but one must note that this supersaturation state is essential to allow the coexistence of this kind of medium with mineralized HAP tissues since, otherwise, HAP could dissolve from mineralized tissues into the serum.

(ii) *Extrinsic factors due to physicochemical conditions*, including pH, T and IS, particularly influence the activities of ions and, thus, the solubility of CaP solids. In biological systems, these conditions are also regulated by homeostasis, which maintains these variables in a physiological balanced state.

(iii) *Extrinsic factors due to the presence of specific molecules* that may promote or inhibit the nucleation and/or the growth of CaP. These include inorganic molecules, such as pyrophosphate (PP_i_), and organic compounds, mainly non-collagenous proteins (NCPs). A major group of non-collagenous proteins present in mineralized tissues is the small integrin-binding ligand N-linked glycoprotein (SIBLING), which is reported to play a pivotal role in the regulation of mineralization processes [52,53,54]. The SIBLING family is composed of five proteins, including osteopontin (OPN), matrix bone sialoprotein (BSP), matrix extracellular phosphoglycoprotein (MEPE), dentin matrix protein 1 (DMP1) and dentin sialophosphoprotein (DSPP). These proteins exhibit a variety of common structural and functional characteristics. First, they are intrinsically disordered proteins (IDPs), which means that they include localized unstructured regions or are globally unstructured. Their discovery disrupted the main paradigm of structural biology according to which the folded protein structure is required for biological function [55,56]. This property confers to the proteins more flexibility, in particular, the remarkable ability to fold/unfold upon binding. Second, they include a collagen-binding domain, a HAP-binding domain, and a cell-membrane-binding RGD motif (Arg-Gly-Asp), which support their involvement in the mineralization processes. Third, they are all submitted to post-translational phosphorylation. Sibling proteins may “promote” or “inhibit” CaP mineralization, presumably within a cooperative process involving several actors (enzymes, collagen, etc.). However, the precise mechanism by which they influence the nucleation and growth of the mineral phase remains elusive, and it is certainly many-sided.

The “inhibitive” role of these proteins is particularly important to explaining the metastability of the biological media regarding HAP precipitation as described in (i). For other proteins such as fetuin, this process has been explained as resulting from the complex formation between biomacromolecules and HAP precursors such as calcium (ii) ions or the sequestration of ACP nanoclusters by phosphopeptides [57]. Indeed, the binding between these molecules and the HAP precursors can be strong enough to block the mineralization kinetically and/or favor the growth according to specific directions. However, one should note that the precise mechanism and the role of these various mineralization key factors may vary significantly. In particular, it must depend on the localization of the processes since the cofactor environment plays an important role in the effect of these molecules.

Accordingly, studying the mechanism of CaP biomineralization requires coping with the complexity of the environment where the mineral nucleates and grows, which is strongly dependent on a variety of physiological factors and biochemical processes of different natures. The development of a biomimetic system may, thus, help to decipher the mechanism by which CaP mineralization is performed in a controlled way in the extracellular matrix (ECM).

The homogeneous versus heterogeneous nature of the mineralization is another important issue that will be tackled hereinafter through the study of biomimetic systems. Indeed, the studies reported until here can be classified as homogeneous processes since the first solid phases nucleate in solution in the absence of seeds (and can act as seeds in the following steps of the mechanism). In the presence of macromolecules, the description of the process is already shifted towards heterogeneous mineralization, as the solids are formed in contact with these molecules, which may decrease their surface energy. This process is then spatially localized. In the next parts, this heterogeneous behavior is even more pronounced, and it is shown to involve specific mechanisms that are not at stake in homogeneous nucleation, such as selective diffusion towards the heterogeneous nucleation site.

## 3. Biogenic Mineralization

In vivo, CaP biomineralization is intimately linked with biomacromolecules, particularly collagen, which is the most abundant protein in mammals [58]. The role of collagen in the mineralization processes has been already pointed out in investigations on the origin of vertebrate skeletons, showing that collagen is used as a template in the fabrication of mineralized tissues [59]. A variety of collagen types, which self-assemble to achieve supramolecular structures with different mechanical requirements, is at the origin of the great diversity of skeletal tissues [60,61,62]. Several collagen types are present in mineralized tissues, such as type I in bone (see below), type X in calcified cartilage [63], etc.

### 3.1. Intrafibrillar Mineralization

#### 3.1.1. Collagen Fibrils

Type I collagen is strongly associated with mineralization processes and has been considered a template in CaP biomineralization, as the scaffold where the mineral formation is initiated [64]. However, this description does not embrace the multiple roles that collagen fibrils play during the biomineralization process, such as specific interaction with non-collagenous proteins [65,66,67] and calcium ions [68]. Type I collagen consists of a right-handed helix composed of three left-handed helix polypeptide chains with nonhelical ends called telopeptides. This yields a highly anisotropic molecular structure with dimensions of ∼300 nm in length and ∼1.5 nm in diameter. In vivo, collagen self-assemble into fibrils via a cell-mediated regulation process (fibrillogenesis) that involves weak intermolecular interactions and covalent bonds. The latter are mainly formed through lysyl oxidase enzymes by catalyzing the deamination of lysine residues, yielding lysine-aldehydes, which then react with hydroxylysine of an adjacent molecule to form a Schiff base [69]. 

#### 3.1.2. Intrafibrillar Mechanisms

Intrafibrillar mineralization consists of the formation of CaP mineral inside collagen fibrils. As previously explained, this mineralization process is often described as involving the formation of ACP during the first step. Once crystallized, the fibrils are expected to contain an assembly of HAP nanoplatelets, and, after that, these mineralized fibrils are supposed to assemble into bigger objects with multi-scale of organization, as described for example in [13].

The very first in vivo observations of intrafibrillar mineralization were made by Jackson in 1957 [70] on thin sections of therso-metatarsus of fowl embryos. This pioneering discovery of 100 Å apatite particles in collagen fibrils was notably confirmed and improved 35 years later by the works of Traub et al. [71] and Landis et al. [72], who both identified potential apatite nucleation sites in collagen fibrils extracted from young turkey tendons. These different studies dealt with young organisms in which the development of mineralized tissues could be more easily observed. Other noteworthy results were obtained by Mahamid et al. in [73] with the study of zebrafish fin rays. Indeed, in this type of organ described as *continuously forming* (and thus always displaying growth zones), the authors evidenced with a set of advanced techniques the presence of 10 to 20 nm ACP particles (yet different from the ACP obtained in vitro) “interspersed around the collagen fibrils” that evolve into carbonated HAP platelets. These studies attracted significantly the attention of the research community onto this specific site of nucleation for HAP, although these investigations remain remarkably rare. This is mainly due to the difficulties of characterizing mineralized tissues at the fibril level while preserving their structure and chemical composition. Indeed, sample preparation procedures usually require a partial demineralization of the tissue. More recent works on mineralized collagen focused on other aspects, such as characterizing the hierarchical organization of bone [13,74].

Intrafibrillar mineralization has attracted a growing interest during the last decades, but many results were obtained for in vitro collagen fibrils. Indeed, state-of-the-art techniques such as highly sophisticated TEM experiments have been performed to finely characterize the mineralization process of in vitro collagen fibrils [75,76] in order to control better the physicochemical state of the studied samples. Then, the intricacy of the mechanism of intrafibrillar mineralization has led to the use of several in vitro models replicating in more or less detail the physicochemical conditions of biomineralization. Many strategies are indeed proposed to describe intrafibrillar mineralization. They highlight several aspects of it.

(1) Most strategies employ collagen fibrils, but some works mimic these scaffolds with other porous materials such as polymer matrices in order to discriminate the confinement effect of the collagen fibrils from the chemical one [25].
(2) In order to prevent extrafibrillar mineralization and to favor it inside the collagen fibrils, most of the studies report the use of NCPs or polyanionic polymers, such as polyaspartic acid [75,77]. These macromolecules are expected to inhibit the mineralization in the surrounding medium and to be size-excluded from the intrafibrillar space (that has been shown to exclude >40 kDa polymers and limit the diffusion of 6 to 40 kDa polymers [78]).
(3) Other strategies to direct mineralization are also described, such as the use of polyelectrolytes. Conceptual models involving nanocluster precursors [20], polymer-induced liquid precursors (PILP) [79] or even both (polyelectrolyte–calcium complex pre-precursor (PCCP) [80]), were proposed to explain the intrafibrillar mineralization due to these polyelectrolytes.

In these different cases, macromolecules such as polyaspartic acid or fetuin allegedly play the role of growth inhibitors and targeting agents toward collagen fibrils. Indeed, such species can confer an overall negative charge to the nanocluster, which can then be attracted by positive areas of the collagen fibrils, such as the gaps within the a-band region of the fibrils [75,81,82]. If the use of polyanions (such as polyaspartate [77]) can indeed be justified by electrostatic interactions, some works report also the effect of a polycation (poly(allylamine) hydrochloride), explained by the more general concept of “balance between osmotic equilibrium and electroneutrality” [76], also referred to as “capillary forces” in [79].

Even if these various strategies and findings deviate sometimes significantly from the physiological conditions, they all underline three key concepts characteristic of intrafibrillar mineralization:

(i) Extrafibrillar mineralization must be prevented or limited through the use of inhibitors.
(ii) HAP precursors (molecules, ions, nanoclusters) must enter into the fibrils; they can do so through passive diffusion or capillary forces or can be guided by specific molecules towards the entrance of fibril gap regions or be attracted by specific sites within the fibrils.
(iii) Once inside the fibrils, the crystallization must be favored, either due to physical confinement only (effect of size-exclusion of the inhibitor and/or templating effect) or also due to the chemical role of collagen (that acts then, for instance, as a nucleating agent).

### 3.2. Matrix Vesicles

Matrix vesicles (MVs) refer to spherical microstructures identified as the initial sites of mineral formation in cartilage, bone and dentine prior to the ECM mineralization. They were originally discovered in 1967 when analyzing cartilage by Anderson [83] and Bonucci [84] independently. The biogenesis of MVs, their characteristics (structure and composition) and, more specifically, the mechanism by which they are involved in CaP mineralization have been the subject of numerous and various articles, which are sometimes controversial (see reviews in [85,86,87,88]). The current understanding of MVs and their biological functions associated with biomineralization has been recently reviewed [89]. MVs are derived from the plasma membrane of mineralizing cells, including osteoblasts, odontoblasts and chondrocytes. However, they exhibit a membrane composition that differs from the parent membrane [90]. Importantly, MVs are enriched with a variety of enzymes, which are involved in the CaP mineralization process in different ways. In particular, three phosphatases are implicated in the production of inorganic phosphate (P_i_) and pyrophosphate (PP_i_) and the regulation of their PP_i_/P_i_ molar concentration ratio. Orphan phosphatase 1 (PHOSPHO1) is present within the vesicles and generates P_i_ from phosphocholine groups [91]. Tissue-nonspecific alkaline phosphatase (TNAP or ALP) and ectonucleotide pyrophosphatase/phosphodiesterase 1 (NPP1) act at the outer surface of MVs: NPP1 converts adenosine 5’-triphosphate (ATP) to PPi and Pi, and ALP mainly hydrolyzes PP_i_ and ATP to produce Pi [92]. A simplified description of the enzymatic reaction pathways has been reported recently by Bottini et al. [89]. In the intravesicular space, the generation of P_i_ by PHOSPHO1 and the transport of Ca^2+^ leads to the nucleation and growth of CaP minerals, as broadly reported in many in vitro tests (for reviews see [85,86,89]). In the extracellular space, the situation seems to be more complex. While the nucleation of the mineral is essentially influenced by the supersaturation of CaP ion precursors, physicochemical conditions and the presence of proteins, its growth is reported to be controlled by the PP_i_/P_i_ molar concentration ratio. PP_i_ is, indeed, a by-product of many metabolic reactions, which acts as an efficient inhibitor of CaP mineralization in different ways [93,94]. The PP_i_/P_i_ ratio is fine-tuned by the coordinated enzymatic activities of NPP1 and ALP [89,95]. The intravesicular accumulation of Ca^2+^ and P_i_ ions leads to the nucleation and growth of CaP-mineralized particles within MVs. The vesicles break either under the hydrolysis of the MV membrane by phospholipases [96] or possibly due to the mechanical stress induced by the mineral growth. This leads to the release of CaP minerals, and most probably precursors, in the extracellular space, presumably in the vicinity of collagen fibrils [97,98,99].

Today, matrix vesicles, including exosomes, ectosomes and apoptotic bodies, have attracted a growing interest owing to their strong involvement in bone mineralization and, importantly, their potential use in therapeutic approaches [100].

### 3.3. “Grey Areas”

**Figure 2:**
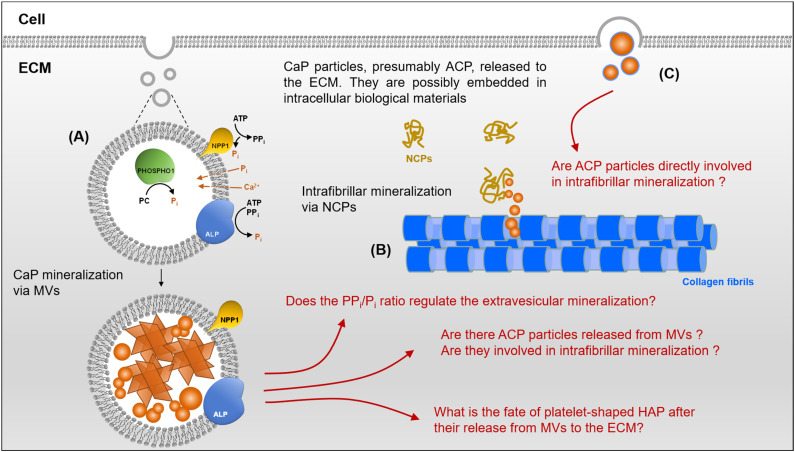
Schematic presentation depicting the “grey areas” regarding the biogenic formation of CaP mineral in the extracellular matrix (ECM). (**A**) Mineralization initiated by matrix vesicles (MVs) by (i) the enzymatic generation of Pi, (ii) the regulation of their P_i_/PP_i_ ratio, (iii) intravesicular accumulation of P_i_ and Ca^2+^ and (iv) formation of ACP mineral (spheroid particles) that crystallize to form HAP platelets. The mechanism of transport of CaP ion precursors, P_i_ and Ca^2+^, indicated by arrows and dashed lines, involves phosphate transporters [101] and annexins [102], respectively, which are not shown. (**B**) Intrafibrillar mineralization mediated by non-collagenous proteins (NCPs). (**C**) Release of presumable ACP particles from the cytosol to the ECM.

The description of the mechanism of CaP biomineralization according to the two proposed views we identified is summarized in Figure 2. It raises a number of issues and shows that the complete picture of the whole process remains unclear. As already described, the extracellular medium does not allow the formation of CaP solid phases. Indeed, even if thermodynamically favorable, it does not happen, most likely due to kinetic considerations promoting the metastability of the system, particularly through the action of NCPs (cf. Section 2.2 (iii)). Then, the first steps of the mineralization of CaP take place in specific localized environments such as MVs or collagen fibrils. This compartmentalization can indeed be seen as a strategy to define the following two regions of space: the main one, which is the extracellular medium where mineralization is inhibited, and localized ones, where mineralization is finely controlled and directed, i.e., within MVs and collagen fibrils.

This well-documented description raises questions that can be summarized as follows:

(i) The accumulation of P_i_ inside MVs occurs through a combination of two different pathways: (1) extravesicular generation of P_i_ involving ALP and NPP1 then trafficking inside MVs through phosphate transporters; (2) intravesicular generation of P_i_ via PHOSPHO1 enzyme. Many studies have shown the necessity of both pathways for the generation of P_i_; otherwise, skeletal mineral deficits or ectopic mineralization can be observed (for a review, see [91]). It is, thus, unclear why both extravesicular and intravesicular generation of P_i_ are necessary, while mineral formation is expected to occur within MVs. Moreover, what happens exactly in the extravesicular space during the mineralization of MVs before their breakdown and subsequent release of CaP minerals? It is, indeed, well-accepted that the growth of CaP mineral outside MVs is controlled by the PP_i_/P_i_ ratio, the role of PP_i_ in bone mineralization being well-documented (see reviews [93,103]). This implies that the mineral also forms in the extravesicular space and that its growth is regulated by PP_i_ and probably by proteins from the ECM. Does this imply then the existence of concomitant intravesicular and extravesicular mineralization processes?

(ii) It has been suggested that CaP minerals form within MVs and grow up to their breakdown, resulting in the release of CaP particles, including HAP crystals, in the extracellular fluid/matrix. The presence of these mineralized particles is, however, difficult to characterize, and their fate in the extracellular space remains unclear. In vitro studies showed that the mineralization within MVs, indeed, leads to the formation of well-defined HAP platelets, clearly visible in the intravesicular space [98]. Even if these platelets are located in the ECM, in the vicinity of collagen fibrils, due to their size, they cannot be loaded within the fibrils, in contrast to the scenario suggested by some authors.

(iii) The above considerations, summarized in (ii), seem to be a complete contradiction with the mechanism of intrafibrillar mineralization, in which the mineral nucleation is supposed to be initiated within collagen fibrils, followed by a growth step that yields HAP platelets inside the fibrils. Although the mechanism involving the intrafibrillar mineralization provides a clear picture regarding the nucleation and growth of CaP particles, it does not explain what happens next in the extrafibrillar space. Indeed, the in vitro models describing intrafibrillar mineralization do not discuss the possibility of a subsequent extrafibrillar mineralization, and, thus, they do not explain the extensive mineralization of the ECM. In bone tissues, the mineral is mostly in the extrafibrillar space; for instance, the amount of mineral outside the fibrils is estimated to represent about 65% of the total mineral phase in the cortex of bovine tibiae [104] and about 60% in Turkey leg tendon [105].

(iv) Another source of CaP precursors has been identified by Boonrungsiman et al. [98]. The authors observed “granules”, presumably made of ACP, residing in mitochondria and transferred to intracellular vesicles, which are then released in the ECM. Recent papers have provided a clearer picture of the transport and fate of these CaP precursors in the intracellular space [106,107]. However, it remains unclear whether the mineral is released in the form of mineral particles or if it is still embedded within the intracellular vesicles. More importantly, and as for MVs, the fate of this mineral in the ECM is not elucidated.

It seems clear that taking into account the intracellular CaP compounds and their transport up to the extracellular space provides a more global view of the mechanism of CaP biomineralization. This points out the multiplicity of the sources of CaP precursors and, thus, the crystallization pathways. However, this does not convincingly elucidate the “grey areas” detailed above. One must keep in mind that in mineralized tissues, such as bone and dentine, collagen fibrils are extensively mineralized in both the intra- and extrafibrillar spaces. Although the mechanisms of mineral formation are very different in these two spaces and involve different actors, it is then difficult to imagine that they operate in independent ways. If mineralization mechanisms, via MVs or intracellular CaP and within collagen fibrils, could, in harmony, explain the formation of the outstanding hybrid systems described above, these pathways need then to accurately coincide in order to ensure the complete mineralization of the ECM and yield these complex hybrid architectures. In other words, if multiple sources of CaP precursors coexist, how do cells regulate HAP crystallization? Such kind of control of the mineralization has, to our knowledge, not yet been described in the literature.

To better investigate the subtle time and space tuning of this balance between extra- and intrafibrillar mineralization, we highlight here the interest of a component that could play a key role in the biomineralization processes: enzymes.

## 4. Enzymatic Approach

As summarized above, the in vivo/in vitro studies describing CaP mineralization processes have shed light upon the precise regulation of mineral nucleation and growth in space and in time. Organisms deploy a toolkit resulting in the regulation of gene expression and in the tailoring of protein functions. Interestingly, despite the high diversity of mineralized tissues in vertebrates, there is considerable overlap in their enzymatic functions that regulate the amount of CaP precursors and inhibitors. Golub published the first review about “enzymes in mineralizing systems” in 1996 [108] and already pointed out the strong involvement of a variety of enzymes in the regulation of CaP mineralization. Through the detailed analysis of the literature, the development of a biomimetic system based on the use of enzymes to assist CaP mineralization appears as a relevant way to enlighten this mechanism in order to reconcile disparate views. Indeed, this simple (from the biological point of view) yet powerful biomimetic system displays many features to explain the phenomena described above:

1/In situ time-controlled generation of calcium phosphate ion precursors. As detailed in Section 2, the formation of ACP and its transformation to HAP are mostly kinetically-controlled processes: time is then a key parameter for these systems, and the temporal evolution of the concentration of the precursors must be carefully monitored and adjusted to suitably mimic biological conditions.

Even if they reproduce the concentrations of ions measured in biological media, the in vitro experiments rely on static descriptions of these systems. Dynamic control of these conditions through the enzymatic release of some important components of the system, such as the calcium phosphate ion precursors, would shed light upon new parameters that could play an important role in the mineralization process. This introduces, indeed, two concepts that are absent in experiments with fixed initial concentrations:

- The introduction of a new kind of supersaturation parameter, σ, of which the time evolution is not necessarily monotonous; it is rather the result of the interplay between the enzyme activity and the kinetics of the mineralization process.
- The dynamics of σ described above can then be controlled by more regulating cofactors, as it results from an enzymatic process, that can a priori be more finely tuned than a continuous addition of calcium phosphate precursors in solution.

2/In addition to temporal control, as described in 1/, the enzymatic system provides also a spatial control for the mineral nucleation that is equally required. Indeed, as mentioned above, compartmentalization and the use of biomacromolecules go naturally hand in hand with the concepts of heterogeneous catalysis that can be obtained with immobilized enzymes. This is particularly relevant to initiate mineralization locally, including in confined media.

3/Moreover, the enzymatic approach presents practical advantages related to the use of purified enzymes. These include the control of the activity by (i) adjusting the concentration of the substrate, (ii) adding metal ions (e.g., Mg^2+^), (iii) modulating physicochemical conditions, (iv) using recombinant enzymes, etc.

A few preliminary results are detailed in the next subsections, depending on whether the enzymes are free in the solution or immobilized on solid supports.

### 4.1. Alkaline Phosphatase

Alkaline phosphatase (ALP, EC 3.1.3.1) activity is the first enzymatic reaction identified in bone by Robinson (1923), who detected the conversion of a phosphoric ester to free phosphate in the femur of a young rabbit [109]. Today, this enzyme is among the most studied proteins in mineralized tissues [110,111,112], and the assessment of its activity has become a standard protocol for the evolution of mineralizing cells during in vitro tests [113]. It catalyzes the dephosphorylation of a phosphate ester group, yielding alcohol and an inorganic phosphate as follows:

R−PO_4_^2−^ + H_2_O → R−OH + HPO_4_^2−^

**Figure 3:**
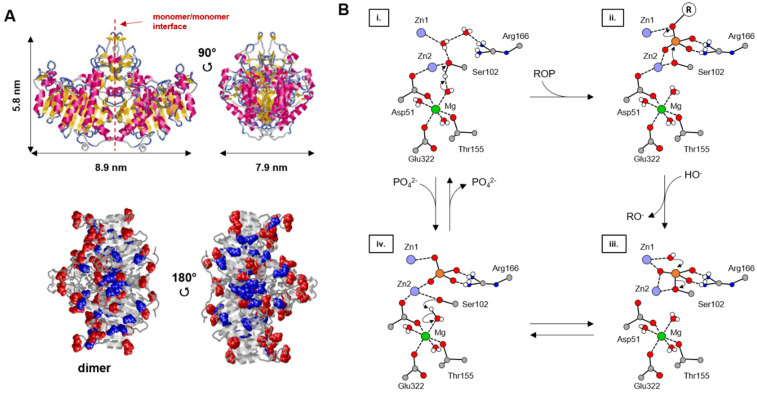
(**A**) Three-dimensional structure of ALP (PDB, code “P19111”). Different views showing the dimensions of the homodimer and the distribution of the superimposed residues, Glu (in blue) and Asp (in red) amino acids (adapted from [118]). (**B**) Mechanism of the enzymatic process (adapted from [117]). Only a few hydrogen atoms have been represented. During the first step, (i → ii), the hydroxide ion bound to the magnesium ion deprotonates the oxygen gamma of Ser102. During the same step, the phosphomonoester substrate (ROP) binds to the two zinc ions and to the guanidinium group of Arg166. Then, during (ii → iii), the deprotonated gamma oxygen of Ser102 acts as the nucleophilic group in the substitution where RO^-^ is the leaving group. Then, during (iii → iv), a hydroxide ion enters the enzymatic site and is coordinated to Zn1. It then attacks the phosphorus atom of the phosphoserine that had been generated in the previous step through a nucleophilic substitution where the gamma alkoxide of Ser102 is the leaving group. Finally, during (iv → i), the alcohol is regenerated on the serine, as well as the hydroxide ion on the magnesium ion. During this last step, a Pi ion is generated.

ALP is present in the form of four isozymes located in different tissues: intestinal, placental, germ cell and tissue non-specific (liver, kidney and bone). In mineralized tissues, mammalian ALP is bound to the outer part of the MV membrane through a glycosylphosphatidylinositol hydrophobic anchor covalently bound to the C-terminus of the enzyme [114,115]. The three-dimensional structure provided by the protein data bank is illustrated in Figure 3A. ALP consists of a dimer with a molecular weight of 57.1 kDa for each monomer. Each chain of the protein exhibits several cofactor binding sites for divalent metal ions: two Zn^2+^ ions and one Mg^2+^ ion, which are both essential for enzymatic activity. The active site was identified as a serine residue located at position 102 (SER 102) of the protein chain [116]. By coordination with serine, the three metal ions are involved in the formation of the nucleophile corresponding to the deprotonated hydroxyl group of serine (Figure 3B(i → ii)). The nucleophile attack can subsequently occur on the phosphorus of the substrate, leading to the formation of a covalent phosphoserine intermediate (Figure 3B(ii → iii)). Then, the Zn^2+^ ion coordinated to serine enables the release of alcohol and the inorganic phosphate [117] (Figure 3B(iv → i)). The latter, generated during the enzymatic reaction, is a competitive inhibitor of ALP that may potentially inactivate the enzyme (Figure 3B(i → iv)). The presence of calcium ions, present during the mineralization, may prevent the enzyme inactivation, as it precipitates with inorganic phosphate at physiological pH.

The cooperativity of mammalian ALP was deeply investigated by Hoylaerts et al., who used wild-type and mutant ALP homodimers and heterodimers [119]. The authors concluded that ALP is a “noncooperative allosteric enzyme”, even if the catalytic property of each monomer is impacted by the conformation of the second subunit.

The research about ALP has exceeded its interest in physiological studies to clarify the role of the enzyme in healthy and pathological mineralization (see for example [111]). The emergence of in vitro enzyme-assisted mineralization has motivated material scientists to explore ALP for the design of CaP-based materials with outstanding mechanical properties and bioactivity [120,121,122,123].

### 4.2. Homogeneous Catalysis

**Figure 4:**
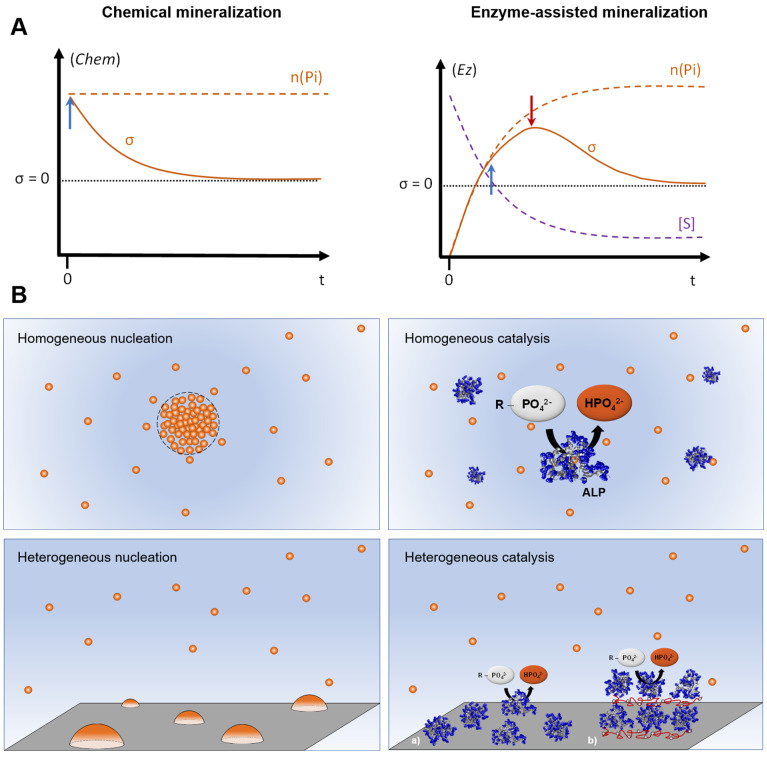
(**A**) Representation of examples of the schematic evolution with time of the supersaturation σ in the presence of an excess of Ca^2+^ ions in the case of (left, *Chem*) chemical mineralization without enzymes or (right, *Ez*) enzyme-assisted mineralization. In the left panel, the phosphate ions (Pi) are generated through the consumption of substrate molecules (S). n(Pi) is the total quantity of Pi introduced in the solution. The blue arrows pointing up (↑) indicate the beginning of the mineralization process (at t = 0 for the case without enzyme), and the red arrow pointing down (↓) represents the moment where the Pi generation through the enzymatic process becomes slower than the Pi consumption due to the mineralization. (**B**) A simplified view depicting (left) chemical vs (right) enzyme-assisted mineralization and the different modes of CaP nucleation. (left, top): Formation of a nucleus from supersaturated solution through a dynamic and stochastic association of ion precursors (spheres), which then grows to mature bulk phase (adapted from [127]). (left, bottom): Heterogeneous nucleation of CaP mineral (hemispherical particles) driven by the surface energy of the support. (Right, top): Enzyme-assisted mineralization through homogeneous catalysis, where the enzyme (ALP) is present in the same phase as its substrate, ester phosphate and CaP ion precursors. (Right, bottom): Heterogeneous catalysis through immobilized enzymes on the support. Immobilization can be achieved by (a) appropriate surface functionalization that favors the interaction with enzyme (physical adsorption or covalent bonds) or (b) by using the sequential layer-by-layer assembly to embed a high number of enzymes and other biomacromolecules of interest. The multilayered film may then serve as a miniaturized bioreactor for mineralization.

In an earlier study [124], we demonstrated that CaP mineralization could be initiated through a homogeneous enzymatic catalysis. Calcium (II) ions and an orthophosphate precursor, namely α-glycerol phosphate magnesium salt, were mixed together with the alkaline phosphatase (ALP) enzyme. With this system, the main difference between a non-enzymatic and an enzymatic system could be compared. Indeed, σ, a key parameter for the mineralization processes, behaves drastically different when comparing systems with an excess of Ca^2+^ with or without an enzymatic generation of the Pi. It is summarized in Section 4 and schematized in Figure 4A, which depicts the evolution of the supersaturation value. In a purely chemical process (without enzyme, *Chem* in Figure 4A), the maximum concentration of Pi is fixed at the very beginning of the experiment. Since it is higher than the value required to trigger the mineralization, this process can start as soon as both Ca^2+^ and Pi are mixed together (t = 0, blue arrow pointing up) and σ decreases continuously until ultimately reaching the saturation value (σ = 1). It is worth noting that in practice, a certain time may elapse prior to the detection of solid particles. This induction time reflects the ability of the solution to maintain a metastable equilibrium state [125,126]. On the contrary, when the Pi is generated through the enzymatic consumption of a substrate S, such as glycerol phosphate [124], σ is initially not defined (*Ez*, Figure 4A). When the substrate is added, σ starts to increase, following the enzymatic kinetic release of Pi until the critical value required to start the mineralization is achieved (blue arrow pointing up). Then, it continues to increase until the speed of Pi consumption through the mineralization process becomes larger than the enzymatic generation rate of Pi (red arrow pointing down). As mentioned in Section 2, as the mineralization of calcium phosphate is a kinetically driven process, these two approaches, *Chem* and *Ez*, can be expected to yield very different results because they involve different mechanisms, as depicted in Figure 4B.

**Figure 5:**
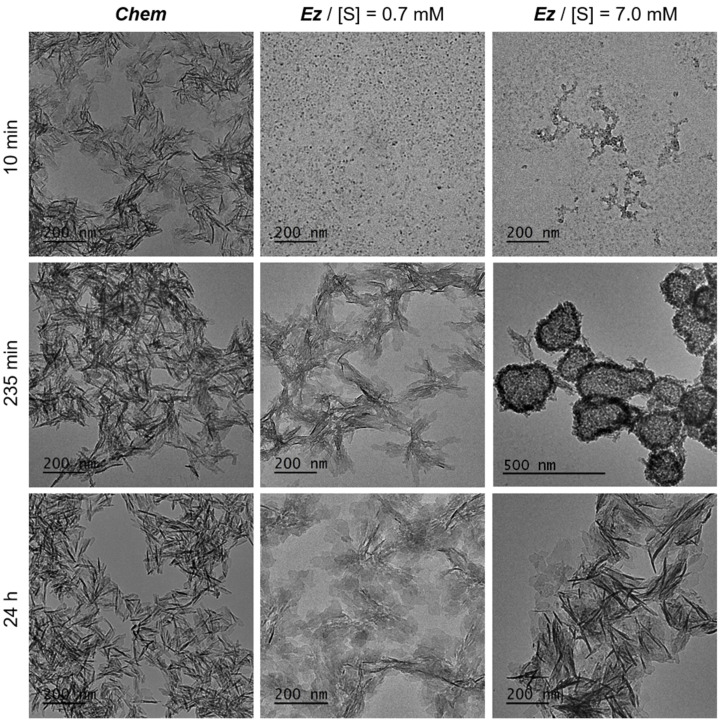
Enzyme-assisted mineralization in homogeneous phase. TEM micrographs for different conditions of mineralization in solution, with [Ca^2+^] = 11.4 mM initially at pH = 7.4 and 37 °C: (left column): without enzyme; (middle column): with ALP enzyme and 0.7 mM of substrate, α-glycerol phosphate magnesium salt (S); (right column): with ALP enzyme and [S] = 7.0 mM. (Top row): minerals obtained after 10 min, (middle row): after 235 min, (bottom row): after 24 h. For [S] = 7.0 mM, after 235 min, DCPD particles, not shown here, were also formed. Adapted from [124].

In Figure 5, TEM micrographs of systems obtained for different initial conditions after various delays are presented as an example of differences between enzymatic and non-enzymatic mineralization, with initial conditions of [Ca^2+^] = 11.4 mM, pH = 7.4 and T = 37 °C. Without enzymes (*Chem*), platelet-shaped particles are already obtained after 10 min and, afterwards, seem to only mature with time, without drastic evolution. With the ALP enzyme, only small clusters (around 10 nm in diameter) are visible after 10 min. They form eventually more or less crystallized platelets but after going through intermediate phases that depend on the initial substrate concentration, [S]. For [S] = 0.7 mM, the objects formed in solution yield poorly crystallized platelets after 235 min that mature slowly with time. For [S] = 7 mM, another type of intermediate can be observed after 235 nm, which displays a rather amorphous core-shell structure from which platelet-shaped particles seem to form. This highlights the interesting influence of the initial substrate concentration on the crystallization pathway followed during the mineralization through the control of the enzymatic activity.

Thus, in [124], various mineralization conditions were explored, and several original results concerning the effect of the enzymatic process were analyzed:

The enzymatic release rate of the orthophosphate (adjusted through the initial enzyme substrate concentration) directly controlled the rate of the mineralization process (followed by real-time dynamic light scattering monitoring).

If the final products of the mineralization were always found to be HAP, according to X-ray diffraction measurements, their dimensions and/or morphology were different (Figure 5). Moreover, unusual intermediate CaP phases, namely DCPD and Whitlockite, were found during the process conducted at 37 °C and at an initial pH of 7.4.

Furthermore, X-ray photoelectron spectroscopy (XPS) measurements revealed the presence of enzymes adsorbed on the CaP solid, suggesting that they could play a role in the mineralization process, and differed depending on the initial conditions.

These findings show that the simple generation of orthophosphate through an enzymatic process can already significantly alter the process usually observed when mixing simultaneously both orthophosphate and Ca^2+^ ions.

### 4.3. Heterogeneous Catalysis

As stated by Golub (1996), “the hallmark of biological mineralization is the precise regulation of mineral deposition in space and time” [108]. The emergence of the concept of enzyme immobilization has offered an outstanding way to preserve, in vitro, various biological activities in space and in time [128]. By immobilizing mineralizing enzymes on solid surfaces, one can combine in situ generation of CaP ion precursor and surface-assisted mineralization, as depicted in Figure 4B, thus offering favorable conditions to direct CaP crystallization through heterogeneous catalysis. The immobilization of enzymes on solid supports may be achieved by using a variety of surface functionalization procedures that allow enzymes to be adsorbed through weak interactions or grafted via covalent bonds depending on the nature of the support [129,130]. The immobilization of a higher number of enzymes may be achieved by embedding enzymes in a multilayered film using the sequential layer-by-layer (LbL) method, originally developed for polyelectrolytes [131]. This approach offers the possibility of incorporating biomacromolecules in thin films while preserving their bioactivity [132] and can be applied to almost all solid supports [133].

#### 4.3.1. 2D Environment

**Figure 6:**
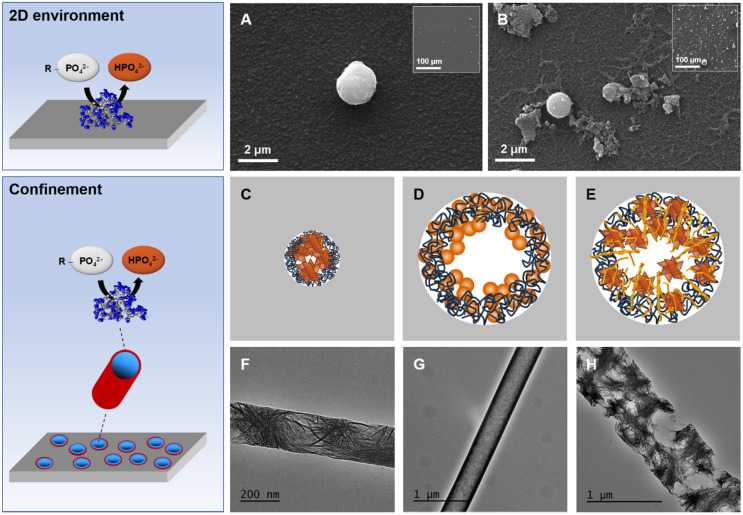
Enzyme-assisted mineralization (top, **A**,**B**) in a 2D environment and (bottom, **C**–**H**) in confinement. SEM images of mineralized LbL film built-up on planar surfaces with (**A**) ALP or (**B**) ALP and type I collagen, both embedded in polyelectrolyte multilayers. (**C**–**E**) Schematic representation depicting the mineralization in a nanoporous template with different pore sizes. (**F**–**H**) TEM micrographs of typical objects obtained after the mineralization for 48 h at pH 7.4 (37 °C) in nanoporous templates with different pore sizes, with or without collagen. (**C**,**F**) The mineralization in 200 nm pore size leads to the formation of nanowires filled with well-crystallized platelet-shaped HAP particles. (**D**,**G**) In 500 nm pore size, the mineralization leads to the formation of spheroid amorphous CaP particles, which grow to form a continuous mineral layer. (**E**,**H**) Embedding type I collagen within the enzyme-based multilayers in 500 nm pore size leads to the formation of HAP crystals stacked onto each other and interconnected with collagen fibrillary nanostructures. Adapted from [134].

In a recent article [118], we immobilized ALP enzymes within a multilayered film, using the sequential LbL technique, and showed the possibility to initiate CaP mineralization on the solid surface. Moreover, by combining real-time monitoring and ex situ characterization techniques, we observed that the growth mechanism of the mineral was significantly impacted by the physicochemical conditions of the medium (pH and temperature). In another study, we embedded type I collagen within a multilayered film containing ALP in a way that mineralization was initiated in the vicinity of self-assembled collagen fibrils [134]. This led to an extensive formation of larger mineral particles, compared to the film without collagen, with higher surface coverage (see Figure 6A,B insets). Additionally, the presence of fibrillar mineralized nanostructures made of collagen and CaP particles was observed by SEM (Figure 6B). These findings confirm the important role that collagen fibrils play in controlling the nucleation and growth of CaP minerals on the solid surface. More importantly, the configuration used in these studies offers a relevant way to mimic the enzymatic generation of Pi within a soft matrix in the vicinity of collagen fibrils.

#### 4.3.2. Confinement

Another factor that organisms exploit to direct CaP crystallization is confinement. The nanoscale confinement has indeed been shown to influence the mineralization kinetics and the mineral morphology, orientation and polymorphism [135]. In practice, carrying out mineralization tests under confinement usually requires the physical separation of CaP ion precursors to avoid precipitation in the bulk. This has been achieved by some authors using a nonporous membrane that separates two compartments containing, respectively, calcium and phosphate ions [136,137]. In this context, the use of enzymes provides, again, a powerful way to direct mineralization in confinement. Recently, we reported the successful immobilization of ALP on the wall of a nanoporous track-etched template using LbL assembly and their subsequent mineralization. This led to the formation of CaP particles along the nanopore wall at different surface coverages and with different morphologies and crystallinities depending on the main factors influencing this process (mineralization time, pH, temperature) [118]. Various results were found for these systems, highlighting the role of the confined generation of orthophosphate to form CaP minerals along the nanopore walls, yielding mineralized nanotube/nanowire objects. Figure 6 summarizes one of the main conclusions of this work. Indeed, a specific trend was found between the diameter of the pores (from 200 to 500 nm) and the crystallinity of the nanotubes: in the smallest pores (200 nm in diameter), well-crystallized platelet-shaped particles were formed (Figure 6C,F), whereas in the largest pores (500 nm in diameter), only amorphous spheroid CaP particles were obtained along the pores (Figure 6D,G). These observations are opposite to the most general trend reported in the literature, showing that increasing the degree of confinement leads to the stabilization of the amorphous phase [136,138]. This discrepancy suggests the existence of a different pathway when the mineralization is initiated by enzymes embedded in polyelectrolyte multilayers. Interestingly, the addition of type I collagen to the studied system induces noticeable effects [134]. For this purpose, collagen was embedded with ALP within the same multilayered film inside the nanopores. The results showed that crystalline platelet-shaped particles were also found in the largest pores (Figure 6E,H), which was not the case in the absence of collagen. This suggests that collagen supplement the lack of confinement and form HAP crystals interconnected by collagen fibrils, as clearly shown in TEM micrographs (Figure 6H), thus enhancing the mechanical stability of these nano-objects.

In conclusion, in these studies, we showed that the localized enzymatic generation of Pi ions could guide the mineralization within the pores of the matrix. It allows benefits from the effect of the confinement within these pores on the formation of crystals that can also be assisted and complemented by the presence of collagen fibrils.

These first results illustrate how the introduction of enzymes in the mineralization process provides a useful tool to guide the formation of CaP crystals. They highlight the crucial role of the control of the mineralization through space and time, which is allowed by the enzyme.

## 5. Prospects/Future Challenges

The hypothesis we developed in this article is the following: through the use of an enzymatic approach, it seems to us that it is possible to unravel new aspects of the mechanism of CaP mineralization. In particular, in our opinion, this approach may reconcile the disparate views of the physicochemistry community (that explores mainly the intrafibrillar mineralization process) with the physiology community (especially dealing with the extracellular matrix vesicles). Broaching the biomineralization issue following an enzymatic approach is a powerful way to provide a better understanding of complex biological mechanisms. The enzymatic approach has been explored in numerous fields, for instance, to unravel the microbial activity of biofilms [139,140], to investigate the mechanism of electron/proton transport involved in the respiratory chain [141,142,143], to assess the xenobiotic metabolism [144], etc.

We think that the addition of enzymes in systems mimicking the biomineralization process is essential since mineralization is not directed solely by the precipitation reaction kinetics. Thus, control mechanisms such as enzymatic kinetics need to also be involved. Our first results in homogeneous phase, from [124], already showed that, while ACP forms initially as in classic in vitro experiments, the crystallization pathway is different compared to abiotic procedures when no enzyme is involved.

Other studies with immobilized enzymes confirmed the complementary role of confinement when associated with localized sources of calcium phosphate precursors.

The addition of more tools from the “biomineralization toolkit” to these in vitro enzymatic model systems, such as mineralization inhibitor proteins or collagen, should give interesting results about the interplay between these various factors and will help to obtain a better description of calcium phosphate biomineralization.

Thus, these findings will doubtlessly provide a better understanding of biogenic CaP formation and the pathologies that can affect it, and they will also allow us to find efficient ways to design new biomaterials to mimic or help to repair mineralized tissues.

## Figures and Tables

**Table 1:** Approximate amount and distribution of calcium and phosphorous in adult humans. Adapted from [49].

	Total Content (g)	Skeleton (%)	Cells (%)	Extracellular Space (%)	Plasma (mM)
**Ca**	1000	>99	<1	<1	2.1–2.6
**P**	700	85	14	<1	0.8–1.5

**Table 2:** Physicochemical characteristics of human plasma. The supersaturation can be defined as: $\sigma =\ln{\left(\frac{IP}{K_{\mathrm{sp}}}\right)}^{\frac{1}{\nu }}$, where *K*_sp_ is the solubility product, *IP* the ionic product, which can be expressed for HAP as $IP=a{\left({\mathrm{Ca}}^{2+}\right)}^{5}\times a{\left({\mathrm{PO}}_{4}{}^{3-}\right)}^{3}\times a\left({\mathrm{HO}}^{-}\right)$, and ν the number of growth unit (here, ν = 9). The activity coefficients at the physiologic condition, computed using the Debye–Hückel law, are $\gamma \left({\mathrm{Ca}}^{2+}\right)$ = 0.27, $\gamma \left({\mathrm{PO}}_{4}^{3-}\right)$ = 0.06, $\gamma \left({\mathrm{HO}}^{-}\right)$ = 0.72.

[Ca^2+^] (mM)	[P_i_] (mM)	T (°C)	*IP*	K_sp_ (HAP)	σ_HAP_
1.1–1.3	0.8–1.5	37	2.4 × 10^−37^	2.35 × 10^−59^	2.4

## Abbreviations

ACP	amorphous calcium phosphate
CaP	calcium phosphate
DCPD	dicalcium phosphate dihydrate
ECM	extracellular matrix
HAP	hydroxyapatite
MVs	matrix vesicles
NCPs	non-collagenous proteins
OCP	octacalcium phosphate
PPi	inorganic pyrophosphate
Pi	inorganic phosphate
